# Clinical Features, Endoscopic Findings, and Predictive Factors for Mortality in Tissue-Invasive Gastrointestinal Cytomegalovirus Disease between Immunocompetent and Immunocompromised Patients

**DOI:** 10.1155/2021/8886525

**Published:** 2021-04-06

**Authors:** Panu Wetwittayakhlang, Natthapat Rujeerapaiboon, Poowadon Wetwittayakhlung, Pimsiri Sripongpun, Nannapat Pruphetkaew, Sawangpong Jandee, Naichaya Chamroonkul, Teerha Piratvisuth

**Affiliations:** ^1^Gastroenterology and Hepatology Unit, Division of Internal Medicine, Faculty of Medicine, Prince of Songkla University, Songkhla 90110, Thailand; ^2^Division of Pathology, Faculty of Medicine, Prince of Songkla University, Songkhla 90110, Thailand; ^3^Epidemiology Unit, Faculty of Medicine, Prince of Songkla University, Songkhla 90110, Thailand; ^4^NKC Institute of Gastroenterology and Hepatology, Songklanagarind Hospital, Prince of Songkla University, Hat Yai, Songkla 90110, Thailand

## Abstract

**Background and Aims:**

Tissue-invasive gastrointestinal cytomegalovirus (TI-GI CMV) disease is common in immunocompromised patients, but the increasing prevalence in immunocompetent patients has been reported. This study compared the clinical manifestations, endoscopic features, treatment outcomes, and predictors for inhospital mortality of TI-GI CMV between immunocompromised and immunocompetent patients.

**Methods:**

Patients with HIV infection, malignancy, or receiving immunosuppressive agents (chemotherapy, high dose, or long-term corticosteroids) were defined as the immunocompromised group. Demographic and inhospital mortality data were obtained and retrospectively analyzed.

**Results:**

A total of 213 patients (89 immunocompetent) with histologically confirmed TI-GI CMV were enrolled. Immunocompetent patients were older (70 vs. 52 years; *p* < 0.001), had more GI bleeding as a presenting symptom (47.2% vs. 29.0%; *p* = 0.010), and shorter symptom onset (2 vs. 14 days, *p* = 0.018). Concomitant extra-GI involvement was only seen in the immunocompromised group (6.5% vs. 0%; *p* = 0.02). Diffuse GI tract (14.5% vs. 4.5%; *p* = 0.032) and esophageal involvement (14.5% vs. 5.6%; *p* = 0.046) were more frequent in the immunocompromised, while small bowel involvement was more frequent in the immunocompetent group (19.1% vs. 8.1%; *p* = 0.029). An overall inhospital mortality was 27.7%. There was no significant difference in inhospital survival probability between the two groups (Peto-Peto test, *p* = 0.65). ICU admission (hazard ratio [HR] 7.21; 95% CI 2.55-20.36), sepsis or shock (HR 1.98; 95% CI 1.08-3.66), malnutrition (HR 2.62; 95% CI 1.05-7.01), and receiving chemotherapy (HR 5.2; 95% CI 1.89-14.29) were independent factors for inhospital mortality. Antiviral treatment for more than 14 days was the only protective factor to improve survival (Peto-Peto test, *p* < 0.001).

**Conclusions:**

Immunocompetent and immunocompromised patients with TI-GI CMV disease had distinct clinical and endoscopic characteristics. There was no significant difference in the inhospital mortality between the two groups. The factors for mortality were ICU admission, sepsis/shock, malnutrition, and receiving chemotherapy. Early diagnosis and initiation of antiviral treatment might improve the survival probability.

## 1. Introduction

The gastrointestinal (GI) tract is the most common site of tissue-invasive cytomegalovirus (TI-CMV) disease [1], defined as the presence of CMV antigens in the immunohistochemistry (IHC) or CMV cytopathic change on the tissue specimen together with symptoms localized to the GI organs [2–6]. The clinical manifestations of the TI-GI CMV disease vary according to GI tract involvement sites [7]. TI-CMV infection is typically found in immunocompromised patients via reactivation of the latent infection [8]. It is associated with high mortality and morbidity, especially in patients with human immunodeficiency virus (HIV) infection, solid organ or stem cell transplantation, immunosuppressive agents, and chemotherapy [9, 10]. CMV infection can be diagnosed by serological or virological detection, but tissue-invasive CMV disease requires histopathological confirmation as a gold standard for the diagnosis [3, 7, 9].

On the other side, TI-GI CMV disease in immunocompetent patients has become more prevalent in recent reports due to increased recognition and improved diagnostic methods [11–18]. CMV becomes reactivated during the phase of immune suppression, mainly in critically ill and elderly patients [14, 19, 20]. Recent studies described the clinical manifestations and associated factors of GI CMV infection in the immunocompetent host [12, 15–18]. However, the comprehensive data of TI-GI CMV disease is relatively limited according to a small number of patients, limited mainly to only CMV colitis, and the diagnosis of TI-CMV disease in some studies had no histopathological proven. Moreover, the benefit of antiviral treatment on mortality in immunocompetent patients remains unclear [21]. Understanding the manifestation and clinical course of TI-GI CMV disease may emphasize an early recognition and improve outcome in the immunocompetent patients.

This study is aimed at comparing the difference between immunocompetent and immunocompromised patients in clinical manifestations, endoscopic findings, risk factors, treatment outcome, inhospital mortality, and predictive factors for survival in the patient with TI-GI CMV disease.

## 2. Materials and Methods

### 2.1. Study Design and Data Collection

We retrospectively reviewed TI-GI CMV patients diagnosed at Songklanagarind Hospital, a tertiary care university hospital and a referral center in Southern Thailand, between July 2005 and March 2020. We identified all adult (age of at least 18 years) patients with histopathological proven TI-GI CMV during the study period from the Division of Pathology database. The information regarding demographic data, comorbidities, medication usage, clinical presentation, endoscopic findings, laboratory data, treatments, and outcomes were obtained from an institutional Hospital Information System (HIS). The characteristics and outcomes of the patients were compared between immunocompromised and immunocompetent groups as the definition described in the next section. This study was approved by the Human Research Ethics Committee (HREC) of the Faculty of Medicine, Prince of Songkla University.

### 2.2. Definitions of Terms

#### 2.2.1. TI-GI CMV Disease

TI-GI CMV disease was defined as an evidence of CMV infection either from (1) typical CMV cytopathic change demonstrated on hematoxylin and eosin (H&E) staining or (2) positive staining for CMV antigen by immunohistochemistry (IHC) in the tissue specimens from gastrointestinal tract (i.e., esophagus, stomach, small intestine, and colon), in which the specimens were obtained from surgical resection or gastrointestinal endoscopy with biopsy. All tissue histology specimens were reviewed by single clinical pathologist (P.W.) who was blinded to clinical characteristics of the patients to confirm the diagnosis of TI-GI CMV before including the patients into the analysis.

#### 2.2.2. Immunocompromised and Immunocompetent Status

All eligible patients were classified into immunocompetent and immunocompromised groups. Patients who were concomitant with the diagnosis of HIV infection, recipients of solid organ or bone marrow transplantation, neutropenia or receiving immunosuppressive agents, i.e., systemic corticosteroid dose ≥20 mg/day of prednisolone or equivalent for more than two weeks, chemotherapy, and immunomodulating agents (e.g., methotrexate, thiopurine, cyclosporine, and tacrolimus) within six months before the time of TI-GI CMV diagnosis were categorized as immunocompromised host. The remaining patients were considered to be in the immunocompetent group, including those with inflammatory bowel disease (IBD) and low dose or a short course corticosteroid administration.

#### 2.2.3. Treatment Outcomes

The treatment outcomes were classified in terms of symptomatic, endoscopic, and histological improvement. The improvement in symptoms was defined as the resolution of the patient's presenting symptoms. The endoscopic improvement was divided into two subcategories: complete or partial endoscopic improvement. Disappearance of all abnormal endoscopic lesions seen on prior endoscopy in the follow-up endoscopy was the definition of complete endoscopic improvement, while the amelioration of the endoscopic findings in comparison with the previous study, e.g., smaller ulcer size or number and resolution of mucosal bleeding, but not completely resolute was characterized as partial improvement. Lastly, the histological improvement was defined by an absence of CMV staining on the follow-up tissue specimen.

### 2.3. Statistical Analysis

Descriptive statistics were used to describe the patient's baseline characteristics. The continuous data were presented as mean ± standard deviation (SD) or median and percentiles (interquartile range [IQR]), while categorical data were expressed as numbers of subjects and percentages. To analyze the difference between two groups of patients, independent *t*-tests and Mann-Whitney *U* test were used to compare the continuous data. The chi-square test or Fisher's exact test was used for categorical data. The inhospital mortality rates were compared using Kaplan–Meier method with the log-rank test or Peto-Peto test. Using the Cox proportional hazard model, the predictors for inhospital mortality and survival were analyzed by univariate analysis, and the multivariate analysis included only the variables with a *p* value of less than 0.2 from the univariate analysis. A *p* value of less than 0.05 was considered to be statistically significant. All statistical analyses were performed using the R program version 3.6.3.

## 3. Results

**Figure 1:**
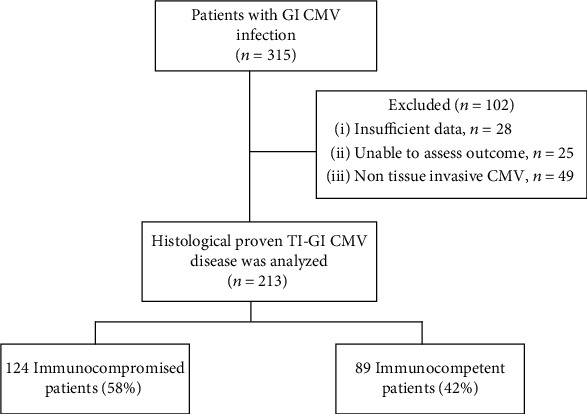
Study flow chart. Abbreviations: CMV: cytomegalovirus; GI: gastrointestinal; TI: tissue invasive.

Of 315 patients with gastrointestinal CMV infection identified by the Division of Pathology database during the study period, 102 patients were excluded from the analysis (Figure 1): 28 patients had insufficient medical information, 25 patients were unable to assess their treatment outcomes due to loss to follow-up or were referred to other hospitals, and 49 patients were considered as a nontissue-invasive form of CMV infection (innocent bystander) as the pathologist deemed neither cytoplasmic change nor uncertain diagnosis after reviewing the tissue specimens. A total of 213 patients were enrolled in the analysis: 124 (58.2%) immunocompromised patients and 89 (41.8%) immunocompetent patients.

Of 124 immunocompromised patients, 34.7% had HIV infection, 38.7% had solid or hematologic malignancies, 5.6% were organ transplant recipients, and 10.5% had neutropenia. The use of systemic corticosteroid, chemotherapy, and other immunosuppressive agents was accounted for 26.6%, 5.6%, and 16.9%, respectively. Among patients with HIV, the median CD4 count was 52 cell/mm^3^ (range 4-226), and 88.4% had a history of opportunistic infections of acquired immune deficiency syndrome (AIDS). For noncorticosteroid immunosuppressive agents, there were thiopurine, methotrexate, cyclophosphamide, tacrolimus, cyclosporin, or other biologic agents used in immunocompromised group.

Comparisons between immunocompetent and immunocompromised patients with TI-GI CMV disease.

### 3.1. Patient Characteristics, Comorbidities, and Clinical Setting

Forty-two percent of TI-GI CMV was immunocompetent patients. The comparison of patients' characteristics between the two groups was demonstrated in Table 1. The patients in the immunocompetent group were significantly older than those in the immunocompromised group (70 vs. 52 years, *p* < 0.001). At the time of diagnosis, the immunocompetent patients were more likely to be diagnosed in the inhospital setting (43.8% vs. 34.7%, *p* < 0.001) and required more intensive care unit admissions (30.3% vs. 9.7%, *p* < 0.001), while the immunocompromised patients were more likely to be diagnosed in outpatient setting (55.6% vs. 25.8%, *p* < 0.001).

As shown in Table 1, the majority of TI-GI CMV immunocompetent patients had advanced age (>65 years old), accounted for 66.3% of patients, 71.9% were in malnutritional status (defined as low body mass index less than 18.5 kg/m^2^ and serum albumin <3.0 g/dL), and 62.9% had CKD of at least stage III. These underlying characteristics were observed significantly more common in immunocompetent than in immunocompromised hosts. Other comorbidities that were also found in the immunocompetent TI-GI CMV more frequently than those with immunocompromised were respiratory failure, bedridden status, and poor controlled DM (defined as HbA1C > 7.0 mg%); although not much prevalent, these features were found significantly higher in the immunocompetent group.

### 3.2. Clinical Presentation

The presenting symptoms were also different between immunocompetent and immunocompromised groups as shown in Table 2. Gastrointestinal bleeding occurred significantly higher in the immunocompetent patients (47.2% vs. 29.0%, *p* = 0.010). Whereas dysphagia or odynophagia (14.5% vs. 4.5%, *p* = 0.032) and significant weight loss (17.7% vs. 3.4%, *p* = 0.003) were more likely to appear as presenting symptoms in the immunocompromised compare to immunocompetent patients. Diarrhea was the most common presenting symptom of TI-GI CMV as it was account for more than a half of patients in both groups. Although the immunocompetent patients had a shorter duration of symptom onset than the immunocompromised patients (2 days vs. 14 days, respectively; *p* = 0.018), the requisite time to diagnosis of TI-GI CMV disease was taken significantly longer in the immunocompetent group compared to the immunocompromised group (19.6 vs. 14.0 days, respectively; *p* = 0.045).

### 3.3. Site of Involvement in TI-GI CMV Disease

To make a definite diagnosis of TI-CMV disease, the tissue specimens were obtained from the index of suspicious site of infection according to the presenting symptoms. The specimens of TI-CMV disease of the patients in this study were attained via esophagogastroduodenoscopy (25.6%), sigmoidoscopy or colonoscopy (62.9%), balloon-assisted enteroscopy (2.4%), or a combination of bidirectional endoscopies (8.3%) and surgery (0.8%). The comparison of the site of involvement and endoscopic finding of TI-GI CMV infection between the two groups is shown in Table 2.

Colon and rectum were the most common sites of involvement in both immunocompetent and immunocompromised groups (66.3% vs. 57.3%, *p* = 0.234, respectively). Interestingly, small intestine involvement was found more frequently in the immunocompetent than the immunocompromised group (19.1% vs. 8.1%, *p* = 0.029), while esophageal involvement (14.5% vs. 5.6%, *p* = 0.046) and diffuse TI-GI CMV infection that involved ≥2 sites of GI tract (14.5% vs. 4.5%, *p* = 0.032) were found significantly higher in the immunocompromised group. Twenty-two patients had more than one site of gastrointestinal CMV infection; 11 patients had colonic with small intestinal involvement (jejunal infection (*n* = 7) and duodenal infection (*n* = 4)); five patients had colonic and gastric involvement; four patients had esophageal and gastric involvement; two patients had gastric and duodenal involvement. Moreover, concurrent CMV infection in extra-GI organ was observed only in the immunocompromised group (6.5% vs. 0%, *p* = 0.022). Of those, five patients were concurrent with CMV retinitis, two patients with pneumonitis, and one patient with CMV meningoencephalitis.

### 3.4. Endoscopic Findings of TI-GI CMV Disease

The ulcerative lesion was the most common finding seen on endoscopies in about two-third of both groups, as shown in Table 2. In more detail, the ulcers appeared as a single lesion in 38% and multiple lesions in 62% of the patients. Deep (54%) and a punch-out ulcer (27%) were the common characteristics of TI-CMV ulcers. The relatively milder lesions, diffuse or focal erythematous, and edematous mucosa were found more frequently in the immunocompromised group than the immunocompetent group (3.4% vs. 14.5%, respectively; *p* = 0.021). In the other findings, erosive lesions or aphthous ulcer (size <5 mm) and diffuse mucosal hemorrhage were found only 12.2% and 6.6%, respectively, whereas the minority of patients had atypical endoscopic findings, which were mass (4.2%) and pseudomembranous lesion (2.3%).

### 3.5. Treatment and Outcome

Of 213 patients with TI-GI CMV infection, 173 patients (81.2%) received antiviral treatment. The immunocompetent group had received antiviral agents in a greater proportion than the immunocompromised group (89.9% vs. 75.0%, respectively, *p* = 0.010), as shown in Table 3. Ganciclovir was prescribed as induction therapy and the first antiviral agent in most treated patients (76.5%), and valganciclovir was used as oral maintenance therapy after induction with ganciclovir in some patients. Only ten patients (16.9%) were treated with valganciclovir as the initial antiviral agents. Antiviral treatment duration was not different between two groups (20.1 days in immunocompromised patients vs. 19 days in immunocompetent hosts, *p* = 0.456).

Among patients receiving antiviral treatment, symptomatic improvement was observed in 127 (73.4%) patients, and there was no significant difference in symptomatic resolution between two groups. Ninety-seven (45.5%) of 213 patients underwent follow-up endoscopy. The time to follow-up endoscopy was not different between the groups. Of those with follow-up endoscopy, the endoscopic findings improved in most of the patients (86.6%). The partial endoscopic improvement was not significantly different between the immunocompetent and immunocompromised groups (53.5% vs. 66.7%, *p* = 0.180); however, the number of patients who achieved complete endoscopic improvement was greater in the immunocompetent compared with the immunocompromised group (32.5% vs. 20.4%, respectively, *p* = 0.048). The tissue samples were taken in all cases who underwent follow-up endoscopy to evaluate the histological improvement, in which more than 80% of histological improvement were observed, and the rate of histological improvement was insignificantly different between the two groups (77.8% vs. 83.7%, *p* = 0.365).

Among the patients who still had detectable CMV infection in the tissue specimens at the follow-up endoscopy, continuation of antiviral treatment was given in 6 of 12 (50%) and 4 of 7 (57.1%), and the resolution of TI-GI CMV disease was subsequently evident in 5 of 6 and all 4 patients in immunocompromised and immunocompetent patients who received the extension of antiviral treatment, respectively. Additionally, the inhospital mortality was not different between the groups, as shown in Table 4.

Two patients developed bowel perforation despite receiving antiviral therapy, which required an emergency surgical treatment. One patient in the immunocompromised group had sigmoid perforation with peritonitis during the fourth day of antiviral therapy. And for the other patient in immunocompetent group, jejunal perforation was detected on abdominal computed tomography as of persistent abdominal pain and fever on the fifth day of antiviral treatment.

Eight patients (3.8%) had recurrent TI-GI CMV disease, defined as a recurrent episode of TI-GI CMV disease after endoscopic and histological resolution of the prior infection. The median follow-up time was 48 months (range 3-76 months), and the median time to recurrence was 5.5 months (range 1.8–27.5 months). Seven of eight patients who had recurrent infection were immunocompromised whereas only one patient in immunocompetent group had recurrent TI-GI CMV disease (5.6% vs. 1.1%, *p* = 0.143).

Among 40 patients who had not received antiviral therapy, the spontaneous improvement in symptoms or endoscopy was observed in 8 (20%) patients during follow-up. The immunocompetent group had a higher rate of spontaneous improvement compared to the immunocompromised group (55% vs. 9.7%, respectively; *p* = 0.035).

### 3.6. Inhospital Mortality and Its Predictive Factors

**Figure 2:**
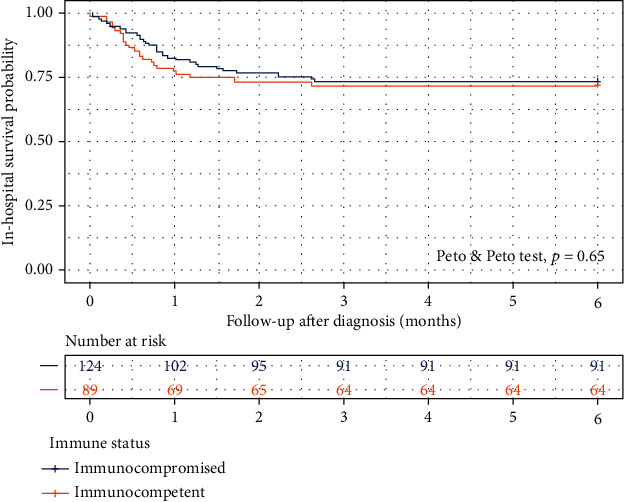
Kaplan–Meier survival curve of inhospital survival probability in immunocompetent and immunocompromised patients.

Fifty-nine (27.7%) of 213 patients died during hospitalization, 34 patients were in the immunocompromised group, and 25 patients were in the immunocompetent group. The inhospital mortality rate was not significantly different between both groups (27.4% vs. 28.1%, *p* = 0.812) during the median hospital stay of 26 days (range 3-184 days). The inhospital survival probability of patients with TI-GI CMV disease was not significant between the immunocompromised and immunocompetent groups (Peto-Peto test, *p* = 0.65) (Figure 2).

The predictive factors for inhospital mortality were analyzed using univariate and multivariate analyses, including age, clinical setting, underlying diseases, comorbidities, and medication use (Table 5). The multivariate analysis revealed 4 factors were independent predictive factors for inhospital mortality, namely, ICU admission (hazard ratio [HR] 7.21, 95% CI 2.55-20.36; *p* < 0.001), receiving chemotherapy, targeted therapy, or immunotherapy (HR 5.2, 95% CI 1.89-14.29; *p* = 0.006), malnutritional status (HR 2.62, 95% CI 1.05-7.01; *p* = 0.040), and presence of sepsis or shock (HR 1.98, 95% CI 1.08-3.66; *p* = 0.025).

**Figure 3:**
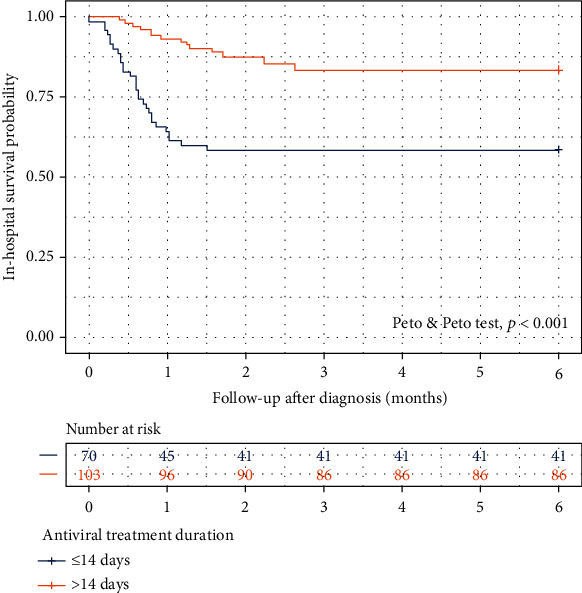
Survival probability of patients receiving antiviral treatment at different duration.

We also determined whether the duration of antiviral treatment affected the in-mortality of the patients. The multivariate analysis showed the antiviral treatment of 15 days or longer was associated with a lower inhospital mortality (HR 0.21, 95% CI 0.09–0.46, *p* < 0.001). We then classified patients into the group receiving antiviral treatment ≤14 days and >14 days. Using the Kaplan–Meier survival curves and Peto-Peto test, the patients who received antiviral treatment more than 14 days had a significantly better survival outcome (Figure 3).

## 4. Discussion

TI-GI CMV infection was generally found in immunocompromised patients. However, the diagnosis of such disease in immunocompetent patients has increased recently due to an accretion of recognition. Our study demonstrated that TI-GI CMV disease in immunocompetent patients was common, accounting for 42% of all TI-GI CMV patients, which is comparable to previous cohorts in which 31%-60% were the infection that had been found in immunocompetent hosts [16, 18].

This study underlined the clinical conditions of the immunocompetent patients, which predisposed them to TI-GI CMV disease. We found that most of TI-GI CMV in immunocompetent patients were elderly (66.3%), being in malnutrition status (71.9%), and had underlying chronic kidney disease (62.9%). Advanced age and multiple comorbidities, especially organ failures, have been previously reported in greater numbers in immunocompetent patients [11, 14–18, 22]. The possible mechanisms are these risk factors are associated with acquired immune dysfunction. The elderly has a weakened immune system, as increasing age is associated with the decline in cellular and humoral immunities, leading to various infections [23, 24]. Patients with renal failure or uremia have impaired immune regulation that suppresses the innate and adaptive immunity to defend gut pathogen and disorder of mucosal immunity from dysbiosis [25].

This study showed that three-fourth of the immunocompetent patient were diagnosed in inpatient setting, and one-third of them required intensive care admission. There was a previous study reported that the reactivation of CMV frequently occurred in 27-33% of critically ill immunocompetent patients [19], since severe illness can cause transient immune paralysis from excessive inflammatory cytokines, and resulting in CMV reactivation [19, 20].

We found that TI-GI CMV infection risk was not increased in patients with inflammatory bowel disease, cirrhosis, low dose, or a short course of systemic corticosteroid use. Although patients with cirrhosis may also have altered immune status, our study showed that it is not a risk factor for TI-GI CMV disease in immunocompetent, similar to the previous cohort [18]. The patients with IBD per se were not a high-risk group for TI-GI CMV disease if they did not receive an immunosuppressive agent or high-dose corticosteroid. The increased risk for CMV colitis was found only in patients with active ulcerative colitis who were on high doses of corticosteroids or immunosuppressive agents [16, 26–29].

Acute GI bleeding, as a presenting symptom, was significantly higher in the immunocompetent compared to immunocompromised patients in our study, which is similar to previous reports [14–18]. CMV can cause endothelial inflammation and vasculitis that lead to mucosal ischemia and subsequent GI bleeding [30, 31], and as the immunocompetent patients who got TI-GI CMV were elderly and had multiple comorbidities, the mucosal healing in such patients might be poor and resulted in more GI bleeding as the clinical presentation.

Compared to the immunocompetent patients, extensive GI tract involvement of TI-GI CMV disease was more prevalent in the immunocompromised patients, as well as a higher risk of relapsed disease [32]. What is more, our study confirms that concurrent extragastrointestinal CMV infection was only seen in immunocompromised patients, akin to the previous cohort [18].

In the immunocompetent patients, symptoms onset was shorter than in immunocompromised patients. This might be from the impaired response of host immunity to defense the viral antigen in HIV-infected patients or immunosuppressive recipients, making their clinical onsets subtle [33]. On the contrary, the requisite time to diagnose TI-GI CMV disease was longer in the immunocompetent patients. We hypothesized that the physicians might have low index of suspicion of CMV infection in immunocompetent patients, making the investigation to identify CMV disease usually delayed.

Most of the endoscopic findings of TI-GI CMV disease were ulcerative lesions, as appeared in most reports [12, 14, 16–18, 31]. There was no pathognomonic feature to aid in the differentiation of TI-GI CMV disease between the groups. Nevertheless, nonspecific lesions, either mucosal erythema or edema, could be the endoscopic manifestation of TI-CMV in the minority of the patients, particularly in immunocompromised group. This highlights the benefit of tissue biopsy to increase the diagnostic yield of TI-CMV even if only nonspecific lesion is observed in the patients with clinical suspicion.

The inhospital mortality rate of the patients with TI-GI CMV disease was not significantly different between the immunocompromised and the immunocompetent groups. The earlier cohorts reported mortality rates ranging from 7.8 to 71% [14–18]. In our study, the overall in-mortality rate was 27%, similar to the report from Le et al. (26%) [17].

The independent predictive factors associated with a higher mortality were critically ill patients who needed ICU admission, patients receiving chemotherapy, malnutrition, and sepsis/shock, respectively. The host immune status (immunocompetent or immunocompromised) was not a significant factor for mortality in our study, in contrast to the previous cohorts [17, 18]. Treatment is generally recommended in TI-CMV disease [4]. According to the current guidelines, ganciclovir is recommended for at least 2–3 weeks in solid organ transplant recipients [9, 34, 35]. Our study found that the duration of antiviral treatment of longer than 14 days was associated with a better survival probability.

The improvement in symptoms, endoscopy, and histology was not significantly different between two groups, though the higher rate of complete endoscopic improvement was observed in the immunocompetent group. We presuppose that the immune response in the immunocompetent group may be more resilient, resulting in faster time of mucosal recovery.

Our study also demonstrated that 55% of the immunocompetent patients who did not receive antiviral treatment had spontaneous TI-GI CMV infection resolution. In comparison, spontaneous resolution occurred in only 9.7% of nontreated immunocompromised patients. The data from a systemic review showed that spontaneous resolution of CMV colitis may occur in immunocompetent with age < 55 years and had no comorbidities [22]; therefore, antiviral agents should be considered in selected immunocompetent individuals.

In this cohort, the immunocompetent patients received antiviral treatment more remarkably than immunocompromised patients as the clinical setting of immunocompetent patients was generally more severe, and the physicians might consider to start treatment easier. In contrast, the immunocompromised patients had fewer symptoms and likely to have a higher risk of adverse effects and drug interaction with ganciclovir [36], as the infection usually occurred in patients with neutropenia, HIV infection, and receiving multiple immunosuppressive agents.

The virtues of our study are as follows: this study reports a large number of patients, comparing TI-GI CMV disease between immunocompetent and immunocompromised groups to demonstrate the comprehensively distinct patient's characteristics, clinical and endoscopic manifestations, and inhospital mortality and its predictive factors. In addition, all the included patients were biopsy-proven and all specimens were reviewed by the pathologist to confirm tissue-invasive CMV disease. Nonetheless, there are some limitations in this study. First, the majority of immunocompetent patients were elderly and had more severe clinical conditions than the immunocompromised patients. The immunocompetent group also received antiviral treatment significantly greater than the immunocompromised group, and these factors could influence the survival outcome. Second, our study did not provide serological CMV testing as well as CMV viral load; however, histological diagnosis remains the gold standard to diagnose CMV disease because CMV viremia is not universal in CMV disease, especially in the GI tract [10, 37]. Third, this study did not perform the quantitative polymerase chain reaction (qPCR) of CMV on endoscopic biopsies since it is a newly developed method. Recent studies showed qPCR might increase the diagnostic yield when using in combination with definitive histopathological diagnosis. Still, we have to be aware of the possibility of nonpathogenic viral shedding [38]. Lastly, according to the retrospective nature of the study, there might be some missing information.

## 5. Conclusion

Immunocompetent and immunocompromised patients with TI-GI CMV disease had distinct clinical and endoscopic characteristics. Immunocompetent patients are older, have more comorbidities, more GI bleeding at presentation, and shorter symptom onset than immunocompromised patients. However, the inhospital mortality was not different. The factors associated with the higher mortality in TI-GI CMV disease were requiring ICU admission, sepsis or shock, malnutritional status, and receiving chemotherapy. Early diagnosis and initiation of antiviral treatment might improve the survival probability.

## Figures and Tables

**Table 1:** Baseline characteristics of TI-GI CMV disease between immunocompromised and immunocompetent patients.

Characteristics	Overall (*n* = 213)	Immunocompromised (*n* = 124)	Immunocompetent (*n* = 89)	*p* value
Age in years, median (IQR)	62 (47-72)	52 (37-67)	70 (63-79)	<0.001
Male gender	134 (62.9)	83 (66.9)	51 (57.3)	0.197
Clinical setting at diagnosis				
Outpatient	92 (43.2)	69 (55.6)	23 (25.8)	<0.001
Inhospital patient.	82 (38.5)	43 (34.7)	39 (43.8)	<0.001
Intensive care unit	39 (18.3)	12 (9.7)	27 (30.3)	<0.001
Requisite time (day) to diagnosis Median (IQR)	16 (8-41)	14 (3-37)	19 (4-41)	0.045
Immunocompromised conditions				
HIV infection	43 (20.2)	43 (34.7)	0	<0.001
Solid malignancy	29 (13.6)	29 (23.4)	0	<0.001
Hematologic malignancy	19 (8.9)	19 (15.3)	0	<0.001
Organ transplantation	7 (3.3)	7 (5.6)	0	0.043
Immunosuppressive agents	21 (9.9)	21 (16.9)	0	<0.001
Neutropenia	14 (6.6)	13 (10.5)	0	0.010
Chemotherapy	7 (3.3)	7 (5.6)	0	0.043
Systemic corticosteroid^†^	33 (15.5)	33 (26.6)	0	<0.001
Comorbidities				
Sepsis	59 (27.7)	29 (23.4)	30 (33.7)	0.132
CKD stage III-IV, ESRD	86 (40.4)	30 (24.2)	56 (62.9)	<0.001
Respiratory failure	59 (27.7)	25 (20.2)	34 (38.2)	0.006
Inflammatory bowel disease	15 (7)	11 (8.9)	4 (4.5)	0.337
Bedridden status	8 (3.8)	0 (0)	8 (9)	<0.001
Cirrhosis	15 (7)	8 (6.5)	7 (7.9)	0.900
Autoimmune diseases.	14 (6.6)	9 (7.3)	5 (5.6)	0.845
Poorly controlled DM	15 (7)	3 (2.4)	12 (13.5)	0.004
Malnutritional status^‡^	131 (61.5)	67 (54.0)	64 (71.9)	0.012
Advanced age (>65 years)	95 (44.6)	36 (29)	59 (66.3)	<0.001
Medication use				
Low-dose corticosteroid	16 (7.5)	7 (5.6)	9 (10.1)	0.339
Exposed antibiotics (>14 days)	37 (17.4)	10 (8.1)	27 (30.3)	<0.001

Data are presented as number (percentage) unless indicated otherwise. ^†^Systemic corticosteroid dose ≥20 mg/day of prednisolone or equivalent for >2 weeks. ^‡^Malnutrition status defined as low body mass index (<18.5 kg/m^2^) and serum albumin <3 g/dL. Abbreviations: HIV: human immunodeficiency virus; CKD: chronic kidney disease; ESRD: end-stage renal disease; DM: diabetes mellitus; IQR: interquartile range.

**Table 2:** The locations and endoscopic finding of TI-GI CMV disease between immunocompromised and immunocompetent patients.

Characteristics	Overall (*n* = 213)	Immunocompromised (*n* = 124)	Immunocompetent (*n* = 89)	*p* value
Presenting symptoms				
Symptom onset in days Median (IQR)	7 (1-84)	14 (1-84)	2 (1-21)	0.018
Gastrointestinal bleeding	78 (36.6)	36 (29.0)	42 (47.2)	0.010
Abdominal pain	45 (21.1)	32 (25.8)	13 (14.6)	0.071
Diarrhea	120 (56.3)	70 (56.5)	50 (56.2)	1.000
Odynophagia or dysphagia	22 (10.3)	18 (14.5)	4 (4.5)	0.032
Nausea/vomiting	7 (3.3)	5 (4.0)	2 (2.2)	0.702
Significant weight loss	25 (11.7)	22 (17.7)	3 (3.4)	0.003
Fever	55 (25.8)	29 (23.4)	26 (29.2)	0.424
Sites of involvement				
Esophagus	23 (10.8)	18 (14.5)	5 (5.6)	0.046
Stomach	11 (5.2)	7 (5.6)	4 (4.5)	0.765
Small bowel	27 (12.7)	10 (8.1)	17 (19.1)	0.029
Colon and rectum	130 (61.0)	71 (57.3)	59 (66.3)	0.234
Diffuse GI lesions (≥2 sites)	22 (10.3)	18 (14.5)	4 (4.5)	0.032
Extra-GI involvement	8 (3.8)	8 (6.5)	0 (0)	0.022
Endoscopic findings				
Erythematous, edematous	21 (9.9)	18 (14.5)	3 (3.4)	0.021
Mucosal hemorrhage	14 (6.6)	5 (4.0)	9 (10.1)	0.137
Erosion	26 (12.2)	13 (10.5)	13 (14.6)	0.487
Ulcer	139 (65.3)	81 (65.3)	58 (65.2)	1.000
Mass	9 (4.2)	6 (4.8)	3 (3.4)	0.738
Pseudomembranous	5 (2.3)	1 (0.8)	4 (4.5)	0.163

Data are presented as number (percentage) unless indicated otherwise. Abbreviations: GI: gastrointestinal; IQR: interquartile range.

**Table 3:** Treatments and outcomes of TI-GI CMV disease between immunocompromised and immunocompetent patients.

Treatment and outcomes	Overall (*n* = 213)	Immunocompromised (*n* = 124)	Immunocompetent (*n* = 89)	*p* value
Antiviral treatment	173 (81.2)	93 (75.0)	80 (89.9)	0.010
Ganciclovir	163 (76.5)	90 (72.6)	73 (82.0)	0.150
Valganciclovir	10 (16.9)	3 (22.6)	7 (0.09)	0.225
Duration of treatment in days, mean (SD)	19.6 (9.7)	20.1 (8.9)	19.0 (10.5)	0.456
Time to follow-up endoscopy in days, mean (SD)	24.7 (7.3)	23.4 (7.4)	26.6 (8.7)	0.673
Symptomatic improvement after antiviral treatment^†^	127 (73.4)	70 (75.3)	57 (71.3)	0.115
Endoscopic follow-up	97 (45.5)	54 (43.5)	43 (48.3)	0.671
Complete improvement^‡^	25 (25.8)	11 (20.4)	14 (32.6)	0.048
Partially improvement^‡^	59 (60.8)	36 (66.7)	23 (53.5)	0.180
Not improvement.	13 (13.4)	7 (13.0)	6 (14.0)	0.725
Histological improvement^‡^	78 (80.4)	42 (77.8)	36 (83.7)	0.365
Perforation	2 (0.9)	1 (0.8)	1 (1.1)	1.000
Recurrence infection	8 (3.8)	7 (5.6)	1 (1.1)	0.143
Inhospital dead	59 (27.7)	34 (27.4)	25 (28.1)	0.812
Spontaneous symptoms and/or endoscopic improvement without antiviral treatment	8 (3.8) (20% of non-treatment)	3 (1.4) (9.7% of non-treatment)	5 (5.6) (55% of non-treatment)	0.035

Data are presented as number (percentage) unless indicated otherwise. ^†^Symptomatic improvement was calculated based on number of patients who received antiviral treatment ^‡^Endoscopic and histological improvement were calculated based on number of patients who underwent follow-up endoscopy.

**Table 4:** Treatment and outcome of CMV-detected patients on the tissue at the follow-up endoscopy.

	Total (*n* = 19)	Immunocompromised (*n* = 12)	Immunocompetent (*n* = 7)	*p* value
Treatment				
Continuation of antiviral treatments	10 (52.6%)	6 (50%)	4 (57.1%)	0.870
No further antiviral treatment/only observative treatment	9 (47.4%)	6 (50%)	3 (42.9%)	0.645
Follow-up outcome				
Improvement on repeated endoscopy and histology	9 (47.4%)	5 (41.7%)	4 (57.1%)	0.730
Inhospital death	8 (42.1%)	6 (50.0%)	2 (28.6%)	0.065
No available data on follow-up/refer to other hospitals	2 (10.5%)	1 (8.3%)	1 (14.3%)	0.900

**Table 5:** Univariate and multivariate analyses (Cox proportional hazard model) to determine the predictive factors for inhospital mortality.

Variable factor	Univariate *p* value	Multivariate^†^ *p* value	Hazard ratio (95% CI)
Immune status	0.722		-
Advanced age (>65 years)	0.045	0.594	0.85 (0.47-1.54)
ICU admission	<0.001	<0.001	7.21 (2.55-20.36)
HIV infection	0.431	-	-
Solid/hematologic cancer	0.003	0.530	0.77 (0.34-1.76)
Immunosuppressive agents	0.225	-	-
Chemotherapy	0.01	0.006	5.2 (1.89-14.29)
Low-dose prednisolone	0.384	-	-
Sepsis/shock	<0.001	0.025	1.98 (1.08-3.66)
CKD/ESRD	0.004	0.299	1.38 (0.75-2.52)
Respiratory failure	<0.001	0.148	1.62 (0.83-3.13)
Cirrhosis	0.472	-	-
Neutropenia	0.059	0.160	2.0 (0.79-5.04)
Bedridden	0.997	-	-
Inflammatory bowel disease	0.537	-	-
Autoimmune diseases	0.923	-	-
Diabetes mellitus	0.087	0.294	1.62 (0.68-3.82)
Malnutrition status	<0.001	0.040	2.62 (1.05-7.01)

^†^Variables with a *p* value less than 0.2 were included for the multivariate analysis using the Cox proportional hazard regression model. Abbreviations: ICU: intensive care unit; HIV: human immunodeficiency virus; CKD: chronic kidney disease; ESRD: end-stage renal disease.

## Data Availability

The data to support the findings of this study are available from the corresponding author upon request.
